# Antisepsis

**Published:** 1896-03

**Authors:** J. M. Spear

**Affiliations:** Cumberland, Md.


ARTICLE IV.

### ANTISEPSIS.

BY J. M. SPEAR, M. D., CUMBERLAND, MD.

Read before the Tri-State Medical Association of Western Maryland, Western Pennsylvania and West Virginia, at Cumberland; Md, December, 12, 1895.

I conceive that the three most important subjects for the railroad surgeon's consideration are, named in the order of their importance, hemorrhage, shock and antisepsis. As the second mentioned subject has been ably discussed at this meeting, and the first mentioned, incidentally, in connection with the subject of shock, I have concluded to make the third the subject of this paper.

While I have admitted that antisepsis occupies third place in importance, I do so under protest and on the grounds that hemorrhage and shock require more activity and energy to avert an impending danger, but, after all, I believe that more deaths occur from a disregard of the principles of antisepsis than would result from all cases of hemorrhage and shock together, though they were left wholly untreated.

As a further excuse for, and as further extenuating circumstances for the crime of offering a paper on so anemic a subject, a subject so well understood and so largely discussed as antisepsis, I must say that I fear many railroad surgeons attach too little importance to its principles, or are careless as to the proper execution of them.

Schwann, in sowing the seed of the germ theory, in the year 1837, made it possible to construct the magnificent superstructure of antiseps's, which has since been built upon that foundation, and it is well for us to ever bear in mind that every detail of antisepsis has for its object the destruction of germs and that asepsis has for its object the protection of the tissues from inoculation with germs.

Long before the recognition of the germ theory we used creosote as a preservative of meats, we used heat as a preservative of fruits, etc., and we used quinine and mercury for their known curative effects in certain diseases, without the knowledge that they all acted as germicides but now, under the searchlight of modern science, we understand their modus operandi, which enables us to apply these and a large number of their rivals more scientifically.

Since carbolic acid volunteered its services, twenty-five or thirty years ago, the ranks of the antiseptic army has greatly swelled and has marched through the medical and surgical field fighting its battles with death and disease, holding the citadel against the pathogenic onslaught until many diseases have almost or quite disappeared from the face of the earth. Hospital gangrene, once the bane of the surgeon, has entirely disappeared, puerperal fever has practically become a disease of the past, the mortality being from all cases of sepsis in four of the largest maternity hospitals in New York City, computed on over four thousand cases, less than one eighth of one per cent., whereas, previous to the introduction of antiseptic precautions in 1883 by Dr. Garrigues, the mortality varied at different periods from five to twenty per cent.

But it is in the field of general surgery that antisepsis finds the widest application. There, too, it has contributed wonderfully to progress. Previous to the last thirty years (antedating the advent of antiseptics) opening one of the large cavities of the trunk or a wound penetrating the brain was looked upon as being almost necessarily fatal, and the opening of a joint was regarded as certainly fatal to the integrity and usefulness of that joint; but now we enter these cavities with impunity and count on perfect motion in the latter cases. So lightly is an exploratory operation of the abdominal cavity regarded by some specialists, that I have heard one say that the operation was no more dangerous than vaccination.

Our experience under the antiseptic regime emboldens us to undertake, successfully, too, operations that the older surgeons would regard as impossibilities. All the different processes of grafting with skin, sponge, muscle, bone, cornea, etc., are only made possible through the educational influence incidental to the introduction of the antiseptic procedure. In the present state of our knowledge it becomes our duty in all cases of fracture of the skull with depression, penetration, hemorrhage, or symptoms of compression, to trephine, elevate, clean and antisepticize, and in all cases of penetration of the larger cavities or severe injury to viscera without penetration it is our duty to enter these cavities, arrest hemorrhage, remove foreign substances, stitch up rents, clean and sterilize in order that the patient may have the best possible chance for his life.

In fractures where the bones cannot be coapted or cannot be maintained in coaptation, it is allowable and proper, under antiseptic precautions, to cut down upon, approximate and retain, by wiring or other mechanical means, the separated fragments. This applies with particular force to fracture of the patella where, although such a process involves the joint, nothing short of drilling and wiring together the fragments promise the best results. It is questionable whether we should not in all cases of fracture or dislocation of the spine, when other means fail to reduce it, cut down upon the injury, remove spiculæ of bone and adjust by sight. This operation, I believe, has been successfully performed by at least one member of our society. Why should it not be tried in all such cases, for can you be summoned to a more hopeless condition than a man with an injury involving displacement of the vertebrae, causing complete paralysis of the parts below? Do you ever feel more helpless in any case?

The suturing together of muscles, ligaments, nerves; and even vessels, have become practical operations, and quite recently an operator has sutured the tendon of one mobile muscle to the tendon of a paralyzed muscle in order to make it do vicarious duty. Such progress is due largely to the beneficient results of antisepsis.

The strict application of Lister's discovery to railroad surgery is the greatest element of conservatism. The lives saved and the functions preserved to parts by it that would have been sacrificed under the old septic treatment cannot be estimated. Neither can the time saved to the employee, as well as to the surgeon, and the money saved to the company by the shorter duration of the benefit period be calculated, to say nothing of the suffering and inconvenience to the patient and his friends.

It is not necessary to add words to what has already been said to prove to this association the benefits and advantages of antisepsis, but it would be time better spent in inquiring how we are to make antisepsis practical in railroad surgery. We must acknowledge that the railroad surgeon labors under great disadvantages. He often has to give the first aid in an open field or may have to perform an important operation in a dirty warehouse, caboose or watchbox. The parts to be treated surgically are always dirty and usually greasy, and the surgeon may find himself limited in other accommodations and appliances without proper assistance.

Under such circumstances the surgeon is apt to excuse himself upon these grounds if he fails to get an aseptic result. It may not always be possible to get the much-desired condition, but with the exercise of good judgment and great painstaking we may generally succeed, and I shall devote the remainder of this paper to a consideration of the means to be employed to reach this coveted goal.

Although the technique of antiseptic surgery, as treated by some authorities, seems quite complicated, it is capable of being reduced to a very simple procedure; and, although there are scores of chemicals employed, one of a selection of three or four of them will be found sufficient to meet any emergency, With iodoform, boracic acid, bichloride of mercury and carbolic acid there is scarcely anything to be desired, unless you class the cleansing agents, such as water, soap, alcohol, ether, etc., along with them. The surgeon should not only be impressed with the idea that cleanliness is godliness, but that it is good surgery and without it we cannot have tolerable surgery.

Cleanliness is cheap, too. Any surgeon can have clean hands, clean instruments and a fair supply of antiseptic dressings with him for all emergency cases. He should have his kit packed and ready to start at a moment's warning. The receptacle need not be fine or expensive. That which I use myself is a cheap canvas telescopic traveling valise. It has the advantage of adapting itself to the bulk of material it is desired to encase. It should contain anesthetics, anodynes, antiseptics, stimulants, instruments, nested enameled pans, towels, ligatures, sutures, bandages and other dressing material, all sterilized, and, what I consider very important, two large bottles, one containing sterile water and the other a standard solution of bichloride of mercury, for you will often be so situated that you cannot procure sterile or even pure water. A few tooth powder or pepper boxes for your antiseptic powders are better than some of the more expensive, and a few feet of half-inch rubber tubing makes the best tourniquets. It matters but little as to the size and weight of your package, within certain limits, because you are generally conveyed to the scene of disaster by rail. So equipped one may feel himself pretty well prepared for any case that is likely to fall to his care, but of course where practicable all serious cases should be removed to a hospital.

The large majority of railroad cases require what might be called only routine surgical treatment. Something like this: The arrest of hemorrhage by simple means, if possible; cleanse the wounds and surrounding parts by water and soap (if necessary to remove grease with kerosene, alcohol or ether) and then with a bichloride solution; perform any necessary operation, again wash with an antiseptic solution and proceed to dress by dusting the parts with iodoform or boracic acid or a mixture of the two; apply aseptic gauze, cotton and bandage. If you have succeeded in suppressing all the hemorrhage, rendered aseptic the parts and have used aseptic absorbable ligatures and sutures this is the only dressing that is likely to be required during the whole process of healing.

My rule for after-treatment following amputations or lacerations is to dress the wound as above indicated, and I seldom find it necessary to disturb it for two or three weeks, at the expiration of such time I usually find it well healed. Of course, general symptoms of hemorrhage may indicate that something has gone amiss and require interference sooner. Above all things avoid adhesive plates and polluted water. Dry dressings have served me best.

The large majority of the injuries we are called upon to treat are not serious, but for that reason it is no less important to give them the best possible treatment, which include strict antisepsis. It makes a vast difference to the company who pays benefits whether the average loss of time of the injured be three weeks, or six weeks, which ratio would about represent the difference between cases treated antiseptically and those treated otherwise. I have seen one after an antiseptic amputation above the elbow return to work in eight days, while one suffering a similar mutilation accompanied by the former usual course of suppuration, would hardly be expected to return to work in as many weeks.

One antiseptic I wish to mention particularly is a chemical combination of gum camphor and carbolic acid in equal proportions. It is well to make the proportion of camphor slightly in excess to be sure that there is no free carbolic acid present. It is not only a powerful antiseptic but is also a powerful local anesthetic as well, and is practically unirritating and non-poisonous withal. Its range of application is wide. It may be applied to mucous or raw surfaces or inflamed integuments. One application will cure ring-worm. I think it would be valuable in rhus toxicodendron poisoning, erysipelas and eczema.

In burns, I have found it a most excellent application, its anodyne properties being not the least important. In extensive burns of all degrees, I have applied it full strength with immediate relief to suffering and without the manifestation of toxic symptoms, but perhaps mixed with six or eight parts of olive oil it would be as effective.

It is insoluble in water, glycerine, or linseed oil, but is a solvent of various chemicals, such as camphor, which it dissolves in large proportion; menthol and chloral, each of which it dissolves in equal parts; iodine, in the proportion of 1 in 30; cocaine, 1 in 10; and morphia, 1 in 30. It is itself soluble in olive oil in the proportion of 1 to 2, and vaseline in the proportion of 1 to 3. In alcohol it dissolves freely.

Now, gentlemen, I would say in conclusion, that, while I have been unable to present anything particularly new on this subject, if I have succeeded in awakening any who are inclined to be lax in antiseptic practice to a realization of their supreme importance, or have been able to point out simple and practical means for their application the object sought for by this paper will have been attained. —*Md. Med. Journal.*

